# The utility of hyperthermia for local recurrence of breast cancer

**DOI:** 10.1186/1477-7819-10-201

**Published:** 2012-09-27

**Authors:** Daigo Yamamoto, Toshio Inui, Yu Tsubota, Noriko Sueoka, Chizuko Yamamoto, Kayoko Kuwana, Mitsuo Yamamoto

**Affiliations:** 1 Department of Surgery, Kansai Medical University, Hirakata, Japan; 2 Iuni Clinic, Moriguchi, Japan; 3 Department of Internal Medicine, Seiko Hospital, Neyagawa, Osaka, Japan

**Keywords:** Breast cancer, Skin metastasis, Hyperthermia

## Abstract

**Background:**

Hyperthermia has long been used in combination with chemotherapy or radiation therapy for the treatment of superficial malignancies, in part due to its sensitizing capabilities. Patients who suffer from superficial recurrences of breast cancer have poor clinical outcomes. Skin metastases may particularly impair the quality of life due to the physical appearance, odor and bleeding.

**Case presentation:**

A 66-year-old woman underwent mastectomy and axillary lymph node dissection for breast cancer. Nine years post-operatively, local metastases developed in the left axillary area (measuring 5 cm in diameter). Initially the tumor did not respond to radiation therapy and chemotherapy. Therefore, we added hyperthermia combined with them. Eight weeks later, the tumor became nearly flat and the patient noted improved activity in her daily life.

**Conclusion:**

Hyperthermia may accelerate the antitumor effects of radiation therapy and chemotherapy. This treatment provides an alternative for unresectable breast cancer skin metastases.

## Background

Hyperthermia has been combined with radiotherapy in an effort to improve local control, which is essential in unresectable locally advanced breast cancer (LABC). Hyperthermia’s ability to affect cells in S phase, inhibit sub-lethal damage repair, and improve oxygenation make it an attractive therapy to combine with radiation and/or chemotherapy in the hopes of synergy [1-5]. The ultimate goal of the addition of hyperthermia to treatment for LABC is improved tumor kill, which most often is assessed with the rate of clinical complete response/partial response (cCR/pPR), and if the patient undergoes surgery, pCR. In addition to the inherent biology of an individual tumor, achieving a CR with thermoradiotherapy depends on the size of the tumor, dose of radiotherapy used, and ability to adequately heat the tumor, which can be especially challenging with large burdens of unresectable disease [6].

## Case presentation

**Figure 1:**
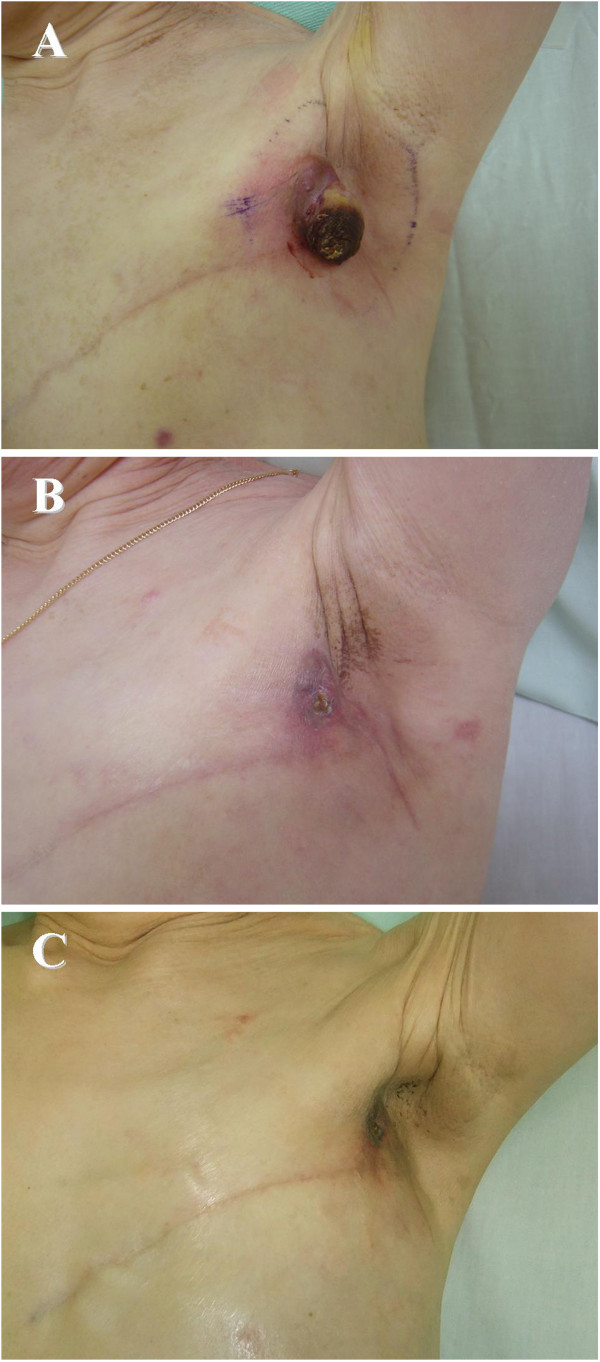
**Skin metastasis from breast cancer was found in the left axillary area.** (**A**) Before hyperthermia. The diameter of the tumor was 5 cm. Four weeks (**B**) and eight weeks (**C**) later, the tumor showed gradual shrinkage.

A 66-year-old woman underwent mastectomy and axillary lymph node dissection for breast cancer. The histopathological findings were papillo-tubular carcinoma with metastases in two axillary lymph nodes. IHC staining showed that estrogen receptor (ER) and progestin receptor (PR) were positive, and Human Epidermal Growth Factor Receptor 2 (HER-2) oncoprotein was negative. As adjuvant therapy, doxorubicin (60 mg/m^2^) and cyclophosphamide (600 mg/m^2^) were administered four times and aromatase inhibitor was prescribed for five years. There was no post-mastectomy radiotherapy (PMRT). Nine years post-operatively, local metastases developed in the left axillary area. Chemotherapy (weekly paclitaxel: 80 mg/m^2^) and radiotherapy (total 30 Gy) were attempted, but the lesions continued to enlarge. On examination, a tumor measuring 5 cm in diameter was found in the left axillar area (Figure 1A). Therefore, we added hyperthermia combined with radiotherapy and chemotherapy. Hyperthermia was performed using a Thermotron RF-8 capacitive heating device (Yamamoto VINITA Co., Ltd., Osaka, Japan at a surface temperature of 38°C to 41°C, once a week for 50 minutes [6]. The output power ranged from 1,300 to 1,400 W. After four weeks, the surface of the tumor began to dissolve, then became gradually necrotic (Figure 1B). Along with a reduction of the tumor size, the foul odor disappeared. Eight weeks later, the tumor became nearly flat and the response was evaluated as a complete response (CR), according to the Response Evaluation Criteria in Solid Tumors Group criteria, and the patient noted improved activity in her daily life (Figure 1C).

## Discussion

Breast cancer is the most common neoplasm to metastasize to the skin. Skin metastases impair activities of daily life due to the physical appearance, odor and bleeding. In the present study, chemotherapy (weekly paclitaxel: 80 mg/m2) and radiotherapy (total 30 Gy) were attempted, but the lesions continued to enlarge. On examination, a tumor measuring 5 cm in diameter was found in the left axillar area (Figure 1A). Therefore, we added hyperthermia combined with the other therapies. In general, the first-line therapy for lymphadenopathy is radiotherapy in which the area was irradiated(total 30 Gy). Thus, we selected hyperthermia, as the radiotherapy (total 30 Gy) was already applied to the area of axillary lymphadenopathy. As a result, we succeeded in controlling these symptoms through the combined application of chemo-radiation therapy and hyperthermia, which resulted in a greatly improved quality of life.

Hyperthermia has been investigated in several randomized and non-randomized clinical trials for cancer therapy [7-11]. Hyperthermia can increase the therapeutic ratio by enhancing the permeability of tumor blood vessels to liposomes. In pre-clinical studies it has been demonstrated that the rate of liposomal extravasation is enhanced four- to eight-fold for temperatures in the target range of this trial [12]. In cats with soft tissue sarcomas, hyperthermia enhanced radio-labeled liposomal uptake by 4- to 16-fold compared to normothermia [13]. Hyperthermia also increases oxygen levels within the tumor, which is critical to the effectiveness of radiation and chemotherapy [14-17]. In a clinical setting, some recent studies [18,19] showed that neoadjuvant chemotherapy combined with hyperthermia is a feasible and well-tolerated treatment strategy in breast cancer patients. In this study [18], 19 of 44 patients were deemed inoperable at the initial assessment. Fourteen of these patients had inflammatory disease. Only five patients were candidates for breast-conserving surgery (BCS). Eight patients elected to have BCS, although 16 were eligible after reassessment following neoadjuvant treatment. At surgery, 32 patients (73%) were found to have axillary lymph node involvement. No patients progressed during neoadjuvant therapy. A complete pathological response was seen in four patients (9%). The combined pathological response was 60% (CR: 9%, and PR: 51%). Therefore, there is a possibility that the addition of HT to preoperative chemotherapy increases cCR and pCR rates more so than chemotherapy alone. In our previous studies, patients with pCR to chemotherapy had a favorable prognosis [20,21]. Thus, the goal of adding hyperthermia to radiotherapy and/or chemotherapy is to increase response rates, and hopefully local control and disease-free survival.

## Conclusions

In conclusion, multidisciplinary therapy, such as hyperthermia, radiotherapy and chemotherapy, may be a useful and effective method for the treatment of progressive breast cancer.

## Consent

Written consent was obtained from the patient for publication of this study and the related photos.

## Abbreviations

BCS: Breast-conserving surgery; cCR: Clinical complete response; CR: Complete response.

## Competing interests

The authors declare that they have no competing interests.

## Authors’ contributions

DY, YT, NS, and KK performed chemotherapy as a team. TI and CY treated the patient using hyperthermia. DY drafted the manuscript. MY carried the data acquisition. All authors read and approved the final manuscript.
